# Potentially inappropriate prescribing in elderly patients with epilepsy at two referral hospitals in Ethiopia

**DOI:** 10.3389/fmed.2024.1403546

**Published:** 2024-08-29

**Authors:** Tamrat Assefa Tadesse, Alemu Belayneh, Minychel Wale Aynalem, Yared Mamushet Yifru, Firehiwot Amare, Dessale Abate Beyene

**Affiliations:** ^1^Department of Pharmacology and Clinical Pharmacy, School of Pharmacy, College of Health Sciences, Addis Ababa University, Addis Ababa, Ethiopia; ^2^Department of Pharmacy, All Africa Leprosy Tuberculosis and Rehabilitation Training Centre Hospital, Addis Ababa, Ethiopia; ^3^Department of Neurology, School of Medicine, College of Health Sciences, Addis Ababa University, Addis Ababa, Ethiopia; ^4^Department of Pharmacy, Asrat Woldeyes Health Science Campus, Debre Berhan University, Debre Birhan, Ethiopia

**Keywords:** epilepsy, elderly, potentially inappropriate prescribing, polypharmacy, Ethiopia

## Abstract

**Introduction:**

The prevalence of drug therapy problems in patients with epilepsy has been reported to be as high as 70–90%. Moreover, elderly patients with epilepsy are highly vulnerable to inappropriate therapies. This study aimed to evaluate potentially inappropriate prescriptions (PIP) in elderly patients with epilepsy at the adult neurology clinics of two referral hospitals in Addis Ababa, Ethiopia.

**Methods:**

A cross-sectional study was conducted on 81 patients with epilepsy and the medication appropriateness index (MAI), the Beers, and Screening Tool of Older Persons’ Prescriptions and Screening Tool to Alert to the Right Treatment (STOPP/START) criteria were used to assess PIP. Data were analyzed using the Statistical Package for the Social Sciences (SPSS) version 25.

**Results:**

Of the 81 study participants, 41(50.6%) were male, and the mean age was 67.33 ± 17.43 years. One-fourth of the study participants (25.9%) had polypharmacy and drug-drug interactions (DDIs) were documented in 64 (79%) patients. Based on the MAI, of the 263 medications that were prescribed for elderly epileptic patients, 110 (41.8%) had drug interactions, 44 (16.7%) had inappropriate indications, 31 (11.8%) were ineffective, and 12 (4.6%) were prescribed incorrect doses. Based on the STOPP and START criteria, PIP was reported in 31(38.3%) and 13(16.1%) patients, respectively.

**Conclusion:**

Polypharmacy and DDIs are common in elderly epilepsy patients. The MAI, Beer’s criteria, and STOPP/START criteria indicate a high prevalence of PIP in elderly patients with epilepsy.

## Introduction

Epilepsy is the fourth most common non-communicable neurological disorder (1, 2). It is characterized by recurrent episodic seizures (3) and is significantly more common in the elderly than in any age group (4). The incidence, prevalence, and mortality associated with epilepsy are all higher in low-income countries than in high-income countries. In particular, the burden of the disease has increased in aging societies (5). The ultimate goal of epilepsy treatment is to prevent and minimize seizure episodes with minimal drug therapy problems (DTPs) and maintain an optimal quality of life through individualized therapy (6). Approximately 50% of patients with epilepsy take at least five medications (polypharmacy), which is three times higher than that in the general population (7). Polypharmacy increases the risk of potentially inappropriate prescribing (PIP), particularly in the elderly (8), and epileptic drugs are associated with many drug interactions that always require assessments when prescribing any medications with them; for example, phenytoin, carbamazepine, and phenobarbital are enzyme inducers that are expected to have drug interactions (9, 10). The prevalence of DTPs in patients with epilepsy has been reported to be as high as 70–90% (6, 11, 12). Moreover, elderly patients with epilepsy are highly vulnerable to potentially inappropriate therapies (11).

The Elderly are usually fragile and more prone to inappropriate medications owing to multimorbidity, polypharmacy, and physiological changes that affect the kinetics and dynamics of drugs (13–16). The prevalence of potentially inappropriate medications (PIMs) varies according to the screening tool used, between 33.9% and 58% in the home context and between 42.4% and 60.5% in hospitalized patients (17). Several factors have been associated with prescriptions in older adults (17). These include increasing age, polypharmacy, diagnosis difficulty, multimorbidity, female sex, dependence on instrumental activities of daily living, frailty, and cognitive impairment. Ultimately, PIMs are associated with an increased risk of adverse reactions and poor patient outcomes (12, 18). A study from an Ethiopian hospital revealed that under-dosing (16.5%), incorrect duration (12.7%), and drug-drug interactions (5%) were the major DTPs among patients with epilepsy (19).

Various tools have been developed to identify potentially inappropriate prescriptions (PIP) in older adults for use in research and clinical settings. These tools can assist physicians with medication management for clinically complex multimorbid older people. Beer’s criteria and STOPP/START) criteria are the most commonly acceptable tools to evaluate PIP and the quality of prescribing among elderly patients (20). In contrast, the Medication Appropriateness Index (MAI) is highly patient-specific and therefore requires not only access to a broad array of clinical data but also the judgment of trained clinicians (21). The STOPP/START criteria (20) and the American Geriatrics Society (AGS) Beers Criteria^®^ are the most widely used tools to address polypharmacy and identify inappropriate prescribing in elderly patients (22). The tools have been associated with positive patient-related outcomes when used as interventions. They are also widely applied and validated internationally in different settings to detect PIMs and potential prescription omissions (22, 23). In Ethiopia, poor medication-related quality of life among older patients was reported by the extent of polypharmacy and PIM use using START/STOPP and Beer’s criteria. A study has been conducted to identify PIP in different diseases (15); however, to our knowledge, there have been no studies or data in Ethiopia that show PIP in older adults with epilepsy. Given the higher number of comorbidities and increased drug burden experienced by this group of patients, we designed this study to evaluate PIP in elderly patients with epilepsy using STOPP/START criteria version 2 and MAI.

## Materials and methods

### Study setting

This study was conducted at the adult neurology clinics of Tikur Anbessa Specialized Hospital (TASH) and Zewditu Memorial Hospital (ZMH) in Addis Ababa, Ethiopia. TASH is a tertiary care specialized hospital that is the largest referral teaching hospital affiliated with the College of Health Sciences at Addis Ababa University. The hospital has approximately 700 beds and serves approximately 1,000,000 patients annually in its outpatient specialty clinics and inpatient and emergency departments. Among the specialty clinics in TASH, neurology provides a comprehensive neurological service to patients with epilepsy, stroke, movement disorders, peripheral neuropathy, and disk prolapse. In the TASH neurology clinic, patients with epilepsy mainly serve on Mondays and Tuesdays. On average, 105 patients were served per week. ZMH has outpatient specialty and subspecialty clinics in different fields of medicine. The general neurology clinic serves approximately 50 patients twice a week.

### Study design and study period

A retrospective study design was employed to review the medical and treatment history of elderly patients with epilepsy who were followed up at the neurology clinics of TASH and ZMH for one year, from August 1, 2021, to July 31, 2022. Data were collected between August 15 and 30, 2022.

### Inclusion and exclusion criteria

Elderly patients with epilepsy who had regular follow-ups and were taking antiepileptic drugs for at least one year were included in the study. Patients with incomplete medical records were excluded from this study.

### Study population and sample size

The study population included all patients (aged ≥ 60 years) with a confirmed diagnosis of epilepsy who visited the TASH and ZMH neurology clinics during the study period. We screened 714 patients with epilepsy, and 81 of them met the inclusion criteria, which included regular follow-ups, being on antiepileptic drugs for at least one year, being 60 years or older, and having complete medical records. These 81 patients were included in the final analysis.

### Study variables

The primary outcome/dependent variable was PIP, and secondary outcome measurements included polypharmacy, drug-drug interactions (DDIs), seizure episodes, adverse drug events, and hospital admission. The independent variables included sociodemographic characteristics (age, sex, and place of residence) and clinical characteristics (comorbidity, type, duration of seizure, and duration of treatment).

### Data collection instrument and management

A semi-structured questionnaire was used to collect sociodemographic, clinical, and medication-related data from the patient records. In addition, Micromedex software was used to assess drug-drug interactions in patients receiving AEDs. Based on Micromedex software classification, the severity of drug interactions was categorized as major, moderate, and minor (24). The Charlson Comorbidity Index (CCI) was also used to calculate patients’ comorbidity score by weighting comorbidities (from 1 to 6) based on adjusted mortality risk or resource utilization, and the sum of all weights yields a single comorbidity score for a patient (23). We used the 2019 updated American Geriatrics Society Beers criteria 20 and STOPP/START criteria to assess PIP (22).

#### American Geriatrics Society (AGS) Beers Criteria Tool

The AGS Beers criteria 2019 updated were used in this study to identify medications to be used with caution in older adults with epilepsy drug-drug interactions, and medication dose adjustment based on renal function. The AGS Beers criteria are an important evidence-based tool that provides an explicit list of PIMs that are best avoided by older adults in most circumstances or specific situations, such as certain diseases or conditions. The AGS Beers criteria include recommendations, quality of evidence, and strength of recommendation for medications that are potentially inappropriate for older adults. These medications should typically be avoided in older adults with certain conditions, medications that should be used with caution, drug-drug interactions, and medication dose adjustments based on renal function (22).

#### The STOPP/START criteria tool

The STOPP/START criteria version 2 used in this study for potentially inappropriate prescribing in the elderly recognizes the dual nature of inappropriate prescribing by including a list of PIMs (STOPP criteria) and potential prescribing omissions (START criteria). The tool has been expanded and updated to minimize inappropriate prescriptions for the elderly. These criteria were based on a recent literature review and consensus validation by a European expert panel. The STOPP/START criteria facilitate improved medication management in older adults with multimorbidities and polypharmacy (25).

### Data quality assurance

Data collectors were trained to create a common understanding of how to collect data and to familiarize them with the data extraction checklist. The validity and reliability of the instrument were tested using 10 % of the total sample size. Based on the results of the pre-test, appropriate corrections were made before data collection.

### Data analysis

Data were analyzed using the Statistical Package for the Social Sciences (SPSS) version 25. Descriptive statistics, such as frequency, median, and range, were used to summarize sociodemographic, clinical, and treatment characteristics and assess the response distribution.

### Definition of terms

Polypharmacy: when a patient is prescribed five or more medications at the time of hospital discharge (26).

Controlled seizure: the patient had not experienced a seizure in the 1 year before the study period (27).

Uncontrolled seizure: The patient had at least one seizure in the 1 year before the study period (27).

Drug-drug interactions: According to Micromedex, DDIs are classified as major (which is life-threatening and requires medical intervention), moderate (which may require medical intervention), and minor (which has a mild effect and often does not require medical intervention) (24).

Charlson Comorbidity Index: Based on the CCI score, the severity of comorbidity was divided into three levels: mild, with CCI scores of 1–2; moderate, with CCI scores of 3–4; and severe, with CCI scores of ≥ 5 (28).

### Ethical considerations

Ethical approval was obtained from the Ethical Review Committee of the School of Pharmacy, College of Health Sciences, Addis Ababa University Ethiopia (ERB SOP/475/14/2022). Informed consent was not sought for the present study because the patients were not interviewed; rather, we obtained their clinical data by reviewing their charts after permission was obtained from the outpatient departments of the study hospitals. For obscurity, personal identifiers were not used during data collection. Confidentiality was assured throughout the study period.

## Results

### Sociodemographic and clinical characteristics of study participants

Of 81 study participants included in the final analysis, 41(50.6%) were male, the mean age was 67.33 ( ± 17.43, range = 60–91) years, and 48.2% were in the age group of 60–64 years. Most patients (72, 88.9%) had at least one comorbidity, and hypertension was the most common, as reported in 46 (56.8%) of patients, followed by cerebrovascular (hemiplegia) events (23, 28.4%). Fifty-four (66.7%) had one or two comorbidities i.e., CCI scores of 1–2 (Table 1).

**TABLE 1:** Sociodemographic and clinical characteristics of elderly people living with epilepsy (PLWE) attending neurology clinics in Addis Ababa, Ethiopia.

Variables	Category	N	%
Sex	Female	40	49.4
	Male	41	50.6
Age in years	60–64	39	48.2
	65–70	18	22.2
	71–79	28	22.2
	80–91	6	7.4
Residence	Addis Ababa	61	75.3
	Out of Addis Ababa	20	24.7
Presence of comorbidities	Yes	72	88.9
	No	9	11.1
CCI Score	1–2(Mild)	54	66.7
	3–4(Moderate)	12	14.8
	≥ 5 Severe)	6	7.4
Comorbidities	Hypertension	46	56.8
	Cerebrovascular (stroke) events	23	28.4
	Diabetes without complications	10	12.3
	Congestive heart failure	9	11.1
	HIV/AIDS	7	8.6
	None-seizure neurologic disorders	7	8.6
	Metastatic solod tumor	5	6.2
	Psychiatry disorders	5	6.2
	Chronic pulmonary disease	4	4.9
	Benign prostatic hyperplasia	4	4.9
	Thyroid disorders	4	4.9
	Prior myocardial infarction	3	3.7
	Cancer without metastases	3	3.7
	Inflammatory bowel disease	3	3.7
	Anemia	3	3.7
	Parkinson disease	3	3.7
	Dementia	2	2.5
	Mild liver disease	2	2.5
	Moderate- to severe- renal disease	2	2.5
	Others*	5	6.2

Other*: peripheral vascular disease, rheumatologic disease, peptic ulcer disease, diabetes with complications, and renal disease.

Generalized tonic-clonic seizures were the most common type of seizures (32, 39.5%), followed by generalized tonic seizures (27, 33.3%). The median time since the diagnosis of epilepsy was 5 years (IQR 3.0-8.5 years), with the majority of patients (53, 65.1%) being diagnosed within five years. Twenty-two (27.2%) patients experienced one to three seizure episodes during the last one-year follow-up, while 29 (35.8%) had uncontrolled seizures and 35 (43.2%) of study participants who experienced seizure onset were over the age of 60 years. Seizures in the elderly were mostly treated (95.1%) with one antiepileptic drug and were controlled in 66.7% of the patients (Table 2). One patient was hospitalized because of a seizure attack caused by discontinuation of phenytoin.

**TABLE 2:** Seizure-related information in elderly PLWE attending neurology clinics in Addis Ababa, Ethiopia.

Variables	Category	N	%
Type of seizure	Generalized tonic-clonic seizure	32	39.5
	Generalized tonic seizure	27	33.3
	Focal seizure with retained awareness	6	7.4
	Focal seizure with a loss of awareness	5	6.2
	Others*	6	7.4
	Generalized clonic seizure	5	6.2
Time since diagnosis of seizure in years	≤ 5	53	65.4
	6–20	19	23.5
	> 20	9	11.1
Onset of epilepsy in years	< 50 years	13	16.0
	50–60 years	33	40.7
	> 60 years	35	43.2
Seizure episodes since last year	1–3	22	27.2
	4–6	4	4.9
	> 6	3	3.7
Number of AEDs prescribed	One	77	95.1
	Two	4	4.9
Status of Seizure	Controlled	52	64.2
	Uncontrolled	29	35.8

Others*: generalized myoclonic seizures, absence seizure, seizures after trauma, stroke, cancer, and HIV/AIDS.

### Polypharmacy and drug-drug interactions

About a quarter of the study participants (25.9%) had polypharmacy with a median of 3.0 (IQR 1.5- 5.0) medications and 110 DDIs were documented in 64 (79%) patients. One-third of the documented DDIs (37%) were the major type of interactions (Table 3). Antiepileptic drugs accounted for 64.54% of the observed DDIs.

**TABLE 3:** Polypharmacy and drug-drug interactions among elderly PLWE attending neurology clinics in Adds Ababa, Ethiopia.

Variables	Description	*N*	%
Number of drugs prescribed including AEDs	< 5	60	74.1
	≥ 5	21	25.9
Drug-Drug interactions	Major	37	33.63
	Moderate	59	53.63
	Minor	14	12.73

Most DDIs of antiepileptic drugs were observed with simvastatin, olanzapine, nifedipine, calcium carbonate/vitamin D, olanzapine, carvedilol, and clonazepam. The main DDIs observed were due to the effect of AEDs on CYP450 enzymes (Table 4).

**TABLE 4:** Examples of major DDIs of antiepileptic drugs.

	DDI	Severity	DDI outcomes
1	Carbamazepine and Simvastatin	Major	Carbamazepine significantly decreases the plasma concentrations of simvastatin and its pharmacologically active acid metabolite.
2	Carbamazepine and olanzapine	Major	Carbamazepine inducers of CYP450 1A2 decrease the plasma concentrations of olanzapine.
3	Phenytoin and Nifedipine	Major	Phenytoin is a potent inducer of CYP450 3A4 that significantly decreases the plasma concentrations of nifedipine.
4	Nifedipine and Phenobarbital	Major	Phenobarbital is a potent inducer of CYP450 3A4 that significantly decreases the plasma concentrations of nifedipine.
5	Calcium carbonate/Vitamin D and Phenytoin	Major	Concurrent administration of calcium carbonate/vitamin D decreases the bioavailability of phenytoin.
6	Olanzapine and Phenytoin	Major	Phenytoin is an inducer of CYP450 1A2 that decreases the plasma concentrations of olanzapine.
8	Carbamazepine and Clonazepam	Major	Carbamazepine may reduce serum clonazepam levels. The risk of CNS-depressant side effects, such as sedation and apathy, may be increased with this combination.

Common drug classes in elderly people living with epilepsy (PLWE).

**FIGURE 1:**
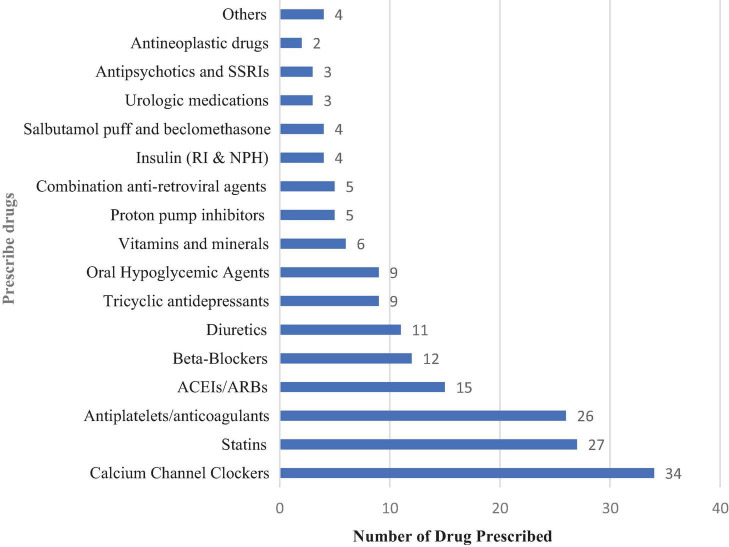
Medications prescribed in elderly PLWE attending the neurology clinics in Addis Ababa, Ethiopia. ACEIs/ARBs, Angiotensin-converting Enzyme inhibitors/angiotensin receptor blockers; RI, regular insulin; NPH, Neutral Protamine Hagedorn; SSRIs, selective serotonin receptor inhibitors; Others, Propylthiouracil, diphenhydramine, levodopa/carbidopa, and erythropoietin.

In this study, 81 elderly PLWE were prescribed 263 medications. Of these medications, calcium channel blockers (12.9%) followed by statins (10.3%) accounted for the largest proportion (Figure 1).

### Antiepileptic drugs in elderly PLWE

**FIGURE 2:**
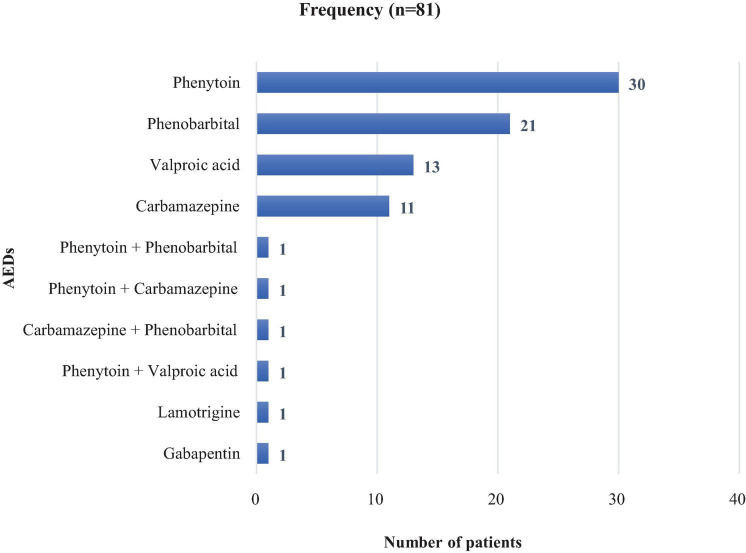
Antiepileptic medications prescribed in elderly PLWE attending the neurology clinics in Addis Ababa, Ethiopia.

Among the AEDs, the highest proportion of prescriptions was for phenytoin, 30 (37.04%) for elderly PLWE, followed by phenobarbitone, 21(21.93%) for elderly PLWE. Only 4 (4.94%) study participants were taking combinations of AEDs (Figure 2).

### Potentially inappropriate prescribing in elderly PLWE according to MAI

According to the medication appropriateness index (MAI), of the 263 medications prescribed for PLWE, 110 (41.8%) had drug interactions, 40 (15.21%) had inappropriate indications, 29 (11.03%) were ineffective, and 11 (4.6%) were prescribed an incorrect dose. Phenobarbitone was the most commonly inappropriately prescribed drug for elderly patients with PLWE, followed by amitriptyline and aspirin. Regarding the effectiveness of the prescribed medications, amlodipine, atorvastatin, and phenobarbitone were the most ineffective prescribed medications in elderly PLWE patients (Table 5).

**TABLE 5:** PIP in elderly PLWE using MAI.

Variables	Frequency
**Not indicated drugs**	
Phenobarbitone	15
Amitriptyline	4
Aspirin	4
Amlodipine	3
Atorvastatin	2
Omeprazole	2
Others^a^	10
**Not effective drugs**	
Amlodipine	4
Atorvastatin	4
Phenobarbitone	4
Amitriptyline	2
Aspirin	2
Nifedipine	2
Omeprazole	2
Simvastatin	2
Others^b^	7
**Incorrectly dosed medications**	
Atorvastatin	3
Simvastatin	2
Others^c^	6

Other^a^: nifedipine, imipramine, carvedilol, diphenhydramine, enalapril, fluoxetine, furosemide, hydrochlorothiazide, regular insulin, and hydroxyurea.

Other^b^: carbamazepine, furosemide, enalapril, hydrochlorothiazide, carvedilol, phenytoin, and valproic acid.

Others^c^: phenytoin, carbamazepine, enalapril, furosemide, phenobarbitone, and fluoxetine.

According to the American Geriatrics Society 2019 Updated AGS Beers Criteria, phenobarbital should be avoided among the elderly but in our study; it was prescribed to 15 (18.52%) patients. Moreover, prescribing AEDs with two or more combinations of antidepressants, antipsychotics, benzodiazepines, or benzodiazepine receptor agonist hypnotics and opioids was observed in three patients. The total PIP prevalence according to the 2019 Updated AGS Beers Criteria was 28.4% (Table 6).

**TABLE 6:** PIP of AEDs in elderly PLWE using Beer’s Criteria.

No	PIP description patients in elderly patients based on Beer’s Criteria	N	%
1	Does the following drug prescribe? Barbiturates (Phenobarbitone)	15	18.5
2	Does benzodiazepines: short and intermediate-acting: prescribed? Lorazepam	1	1.2
3	Does benzodiazepines: long-acting is prescribed? Chlordiazepoxide (alone or in combination with amitriptyline, Clonazepam, Diazepam)	1	1.2
4	Does the following drug prescribe for Absence Seizure? (Carbamazepine, Gabapentin, Phenytoin, Pregabalin)	1	1.2
5	Does antiepileptic medication prescribed with two or more of the following drugs? (Antidepressants (TCAs, SSRIs, and SNRIs) Antipsychotics, Benzodiazepines, Benzodiazepine receptor agonist hypnotics, and Opioids)	3	3.7
6	Gabapentin use with Crcl < 60 (need dose reduction)	1	1.2
7	Pregabalin use with Crcl < 60 (need dose reduction)	1	1.2

### PIP in elderly PLWE using STOPP/START criteria

Based on the STOPP criteria, PIP was reported in 31(38.3%) patients, and medications prescribed without an evidence-based clinical indication were documented in 12(14.8%) patients, followed by drugs prescribed beyond the recommended duration 7 (8.6%) (Table 7).

**TABLE 7:** Potentially inappropriate Prescribing in elderly PLWE using the STOPP tool.

No	PIP description to use in patients in elderly patients based on STOPP criteria	N	%
1	Any drug prescribed without evidence-based indication	12	14.8
2	Any drug prescribed beyond the recommended duration, where treatment duration is well-defined	7	8.6
3	Beta-blocker with bradycardia ( < 50/min), type II heart block, or complete heart block (risk of complete heart block, asystole)	1	1.2
4	Loop diuretic for dependent ankle edema without clinical, biochemical evidence, or radiological evidence of heart failure, liver failure, nephrotic syndrome, or renal failure (leg elevation and/or compression hosiery is usually more appropriate)	1	1.2
5	Thiazide diuretic with current significant hypokalemia (i.e., serum K^+^ < 3.0 mmol/l), hyponatremia (i.e., serum Na^+^ < 130 mmol/l) hypercalcemia (i.e., corrected serum Ca^2+^> 2.65 mmol/l) or with a history of gout (hypokalemia, hyponatremia, hypercalcemia, and gout can be precipitated by thiazide diuretic)	1	1.2
6	Aspirin with a history of peptic ulcer disease without concomitant PPI (risk of recurrent peptic ulcer)	1	1.2
7	PPI for uncomplicated peptic ulcer disease or erosive peptic esophagitis at full therapeutic dosage form > 8 weeks (dose reduction or earlier discontinuation indicated)	3	3.7
8	NSAID with severe hypertension (risk of exacerbation of hypertension) or severe heart failure (risk of exacerbation of heart failure)	1	1.2
9	Vasodilator drugs (e.g., alpha-1 receptor blockers (alfuzosin, doxazosin, terazosin, CCBs, long-acting nitrates, ACEIs, ARBs) with persistent postural hypotension i.e., recurrent drop in systolic blood pressure ≥ 20mmHg (risk of syncope, falls)	3	3.7
10	Dose Indomethacin and ketorolac prescribed?”	1	1.2

According to the START criteria, the prevalence of PIP was 16.05% and was mainly observed with cardiovascular drugs, including antiplatelet 4 (4.9%) and statin 3 (3.7%) therapies (Table 8).

**TABLE 8:** Prevalence of PIMs during hospital visits identified by START.

No	Prescriptions/drug therapies should be considered where omitted for no valid clinical reason(s) in elderly patients based on START.	N	%
1	Antiplatelet therapy (aspirin or clopidogrel) with a documented history of coronary, cerebral, or peripheral vascular disease.	4	4.9
2	Statin therapy with a documented history of coronary, cerebral, or peripheral vascular disease, unless the patient’s status is end-of-life or age is > 85 years.	3	3.7
3	Antihypertensive therapy where SBP consistently > 160 mmHg and/or DBP consistently > 90 mmHg; if SBP > 140 mmHg and/or DBP > 90 mmHg, if diabetic.	2	2.5
4	Regular inhaled beta-2 agonist or antimuscarinic bronchodilator (e.g., ipratropium, tiotropium) for mild to moderate asthma or chronic obstructive pulmonary disease.	1	1.2
5	Regular inhaled corticosteroids for moderate-severe asthma or COPD, where FEV1 > 50% of predicted value and repeated exacerbations requiring treatment with oral corticosteroids.	1	1.2
6	L-DOPA or a dopamine agonist in idiopathic Parkinson’s disease with functional impairment and resultant disability	1	1.2
7	High-potency opioids in moderate-severe pain, where paracetamol, NSAIDs, or low-potency opioids are not appropriate to the pain severity or have been ineffective.	1	1.2

## Discussion

Elderly PLWE are highly vulnerable to PIP because of multimorbidity, polypharmacy, and physiological changes that affect the kinetics and dynamics of drugs, for which a structured approach is recommended to ensure the appropriateness of the prescription (7, 11, 13). Polypharmacy is three times higher in epileptic patients with epilepsy than in the general population (7). This is associated with lower quality of life, increased morbidity, mortality, hospitalization, and increased health care costs (13). Therefore, it is important to optimize drug prescriptions for elderly patients with epilepsy. In this study, one-fourth 21(25.9%) of the study participants had polypharmacy, which is higher than other study findings in Addis Ababa, which reported 10.8% polypharmacy among geriatric patients (29). This might be because the current study was conducted in a tertiary care hospital where complicated cases requiring polytherapy were managed. However, the findings of the current study were lower than those of a study from the United States of America that reported 47% polypharmacy among epileptic patients (7). New clinical evidence advocates the use of carbamazepine, lamotrigine, and other newly developed AEDs rather than phenytoin and phenobarbital as first-line AEDs for newly diagnosed geriatric patients (11). On the contrary, phenytoin was prescribed in a higher proportion of PLWE followed by phenobarbitone. Although phenobarbitone is not recommended for the geriatric population (20), it was the second-most prescribed AED in this study. This could be due to the limited number of safe and effective alternative AEDs in Ethiopia.

The results of this study showed a high prevalence of PIP among elderly epileptic outpatients, which was 28.4% and 38.3%, according to the Beers and STOPP criteria, respectively. This finding is comparable with the finding of a systematic review that reported PIMs in Ethiopia to be (37%), which is the highest PIMs prevalence compared to other developing countries (12). The result of the current study is also similar to another review in Eastern Europe, which reported (34.6%) of PIP (30). The finding of PIP (28.4%) according to Beers criteria is also similar to another study conducted in Nigeria among elderly patients (26.5%) PIP and lower than China studies, which reported 34.39% PIMs among elderly adults (31, 32). But the current study found higher PIP than studies conducted in Jordan and Kuwait, which reported 9.8% and 15.6% PIP, respectively, with CNS medication using the Beers criteria (33, 34). Based on the STOPP criteria, 38.3% of the PIP cases were reported in the present study. A cross-sectional study conducted among older adults with chronic diseases in Ethiopia reported a PIP rate of 45.2%, which was comparable to that in our study. However, studies in Nigeria and Kuwait showed 57.1% and 55.7% PIP, respectively, which is higher than that in the current study (33, 35). The high prevalence of PIP in the current study highlights the need to apply the above criteria in a clinical setting to prevent DTPs in the elderly.

According to the START criteria, 16.05% of prescription omissions were identified. This finding was comparable to that of another cross-sectional study conducted in Kuwait, which reported a 19.8% potential prescription omission (35). In this study, most of the medication omissions were due to cardiovascular medications in patients with cardiovascular comorbidities and epilepsy. Antiplatelet medications and statins were commonly missed. This could be due to documentation omission among patients who had private follow-ups for cardiovascular disease. Miscommunication among different clinics may lead to false medication omission. There was lower medication omission reported in this study than in another study conducted in Jima, Southwest Ethiopia (13). This could be due to the presence of a centralized I-care documentation system for all outpatient clinics in TASH, which shows all medications prescribed from all outpatient clinics. This prevents false prescription omission due to lack of documentation.

In this study, phenobarbitone had the highest prevalence of PIP according to both the Beers and STOP/START criteria. However, According to the American Geriatrics Society 2019 Updated AGS Beers Criteria, phenobarbital should be avoided among the elderly due to the high rate of physical dependence, tolerance to sleep benefits, and greater risk of overdose at low dosages (22). Although phenobarbitone had the above safety concern among geriatric patients, it was the second most commonly prescribed antiepileptic medication following phenytoin in this study, which was prescribed for approximately one-third of 21 (25.9%) study participants. This may be because safer antiepileptic drug alternatives for the elderly are not available in the country.

According to the MAI of 263 medications prescribed for PLWE, 117 (44.5%) were potentially inappropriate. In this study, drug interactions had the highest PIP as identified using the MAI tool. This could be because of the strong potential effects of antiepileptic medications on the CYP450 system. Drug-drug interactions in the present study were lower than those in other studies conducted in Kuwait, Denmark, Sweden, and Australia based on MAI of 73.6%, 94.3%, 63%, and 99% respectively (36–39). On the other hand, the prevalence of PIP with the MAI was lower than the prevalence with the other criteria used in the study. This might be because the MAI does not guide pharmacists to modify medication regimens to avoid potential adverse drug events or add potentially important medications. Clinical pharmacists’ clinical experience may affect the potential for PIMs identification since only clinical pharmacists participated in the assessment of PIMs. However, MAI cannot be identified using medication because it focuses only on prescribed medications. However, one advantage of the MAI is that it can address all medications prescribed to each study participant. Interventions to decrease PIP may contribute to minimizing polypharmacy and drug-drug interactions and optimizing treatment outcomes among elderly patients with epilepsy.

Finally, this study has certain limitations. Data were obtained retrospectively from medical records, and this study design limited the determination of causal relationships among variables and the determination of the etiologies of epilepsy. In addition, we did not use power analysis for the sample size calculation, and the sample size was small because of the rarity of the case. This highlights the need for future research with repeated measures of PIP.

## Conclusion

Most patients were on monotherapy with antiepileptic medications and experienced controlled seizures. Polypharmacy and DDIs are common in elderly epilepsy patients. The MAI, Beer’s criteria, and START/STOP criteria indicate a high prevalence of PIP in elderly patients with epilepsy.

## Data Availability

The raw data supporting the conclusions of this article will be made available by the authors, without undue reservation.
